# Restless legs syndrome: causes and consequences

**DOI:** 10.1007/s00415-019-09682-6

**Published:** 2020-01-07

**Authors:** T. H. Massey, N. P. Robertson

**Affiliations:** Department of Neurology, Division of Psychological Medicine and Clinical Neuroscience, Cardiff University, University Hospital of Wales, Heath Park, Cardiff, CF14 4XN UK

## Introduction

Restless legs syndrome (RLS) of a severity requiring medical attention has a prevalence of 2–3% in Caucasian populations. This significant cause of neurological morbidity is defined by an insistent urge to move the legs, and is often accompanied by uncomfortable and unpleasant sensations deep in the legs themselves. Symptoms typically begin or worsen at rest, especially in the evening and night, and are often relieved by activities such as rubbing, stretching, or walking. There is a family history of RLS in approximately 50% of cases, suggesting genetic risk factors, and significant associations have been shown with low serum ferritin, uraemia, and pregnancy. In addition, individuals with various chronic medical conditions such as cardiovascular disease, hypertension, multiple sclerosis, Parkinson’s disease, spinal cord disease, and neuropathy have been shown to have an increased risk of RLS although proving cause-and-effect is difficult. People with RLS have impaired sleep, often associated with periodic limb movements, and increased risk of depression and anxiety, all of which combine to reduce quality of life. Treatment of RLS involves lifestyle changes, such as increased exercise, and management of chronic comorbidities. Reversible causes such as low serum ferritin should be corrected. Effective medications include dopamine agonists (pramipexole or ropinirole), α2δ agonists (gabapentin or pregabalin), or opioids. RLS symptoms can paradoxically worsen with extended use of dopamine agonists (‘augmentation’) leading to a difficult clinical situation where dopamine agonist withdrawal is required, often with opioid cover.

This month’s journal club examines three papers looking at the causes and consequences of RLS. The first is a meta-analysis of genome-wide association studies that highlight genetic risk factors for RLS. The second and third papers demonstrate associations between RLS and suicide or self-harm and resistant hypertension, respectively, and highlight some of the significant morbidities linked to RLS.

## Identification of novel risk loci for restless legs syndrome in genome-wide association studies in individuals of European ancestry: a meta-analysis

Family studies have estimated the heritability of RLS as 50–60%, suggesting that there is a strong genetic component to disease risk. This risk is polygenic in nature with multiple loci contributing small amounts to the overall RLS risk of an individual. This paper combined three large genome-wide association study (GWAS) data sets of RLS cases and controls into a meta-analysis to identify novel risk loci. The three data sets all comprised individuals of European ancestry, but were recruited in different ways. One was from the EU-RLS-GENE consortium (Europe, Canada, USA) where RLS status was determined by a Neurologist in line with accepted international criteria (6228 cases, 10,992 controls), the second from the INTERVAL study of blood donors in the UK where a validated RLS questionnaire was used to make diagnoses (3065 cases, 24,923 controls), and the third from the commercial genomics company 23andMe where RLS status was determined by the single self-reported response to the question “Have you ever been diagnosed with restless legs syndrome?” (36,603 cases, 346,723 controls).

Meta-analysis of GWAS summary statistics from a discovery data set comprising the EU-RLS-GENE and INTERVAL participants and approximately one-sixth of the 23andMe individuals found 20 independent signals that were significantly associated with RLS. These mapped to 19 independent genomic loci, 13 of which were novel, and were all recapitulated in the replication data set that consisted of the remaining 23andMe individuals. Pathway analyses found that GWAS signals were significantly more likely to be found in genes associated with neurodevelopment, locomotor behaviour, and DNA maintenance and repair. The most significant single-nucleotide polymorphism (SNP) was found in *MEIS1*, a gene involved in controlling HOX gene expression and specifying neuronal cell type. This SNP has a minor allele frequency of 7% and confers a near doubling of risk of RLS in an individual carrying it [odds ratio = 1.92 (1.85–1.99; 95% confidence interval)].

*Comment*. This large meta-analysis confirmed that RLS is a polygenic disorder with multiple relatively common SNPs each conferring small amounts of risk. It identified novel risk loci and biological pathways for further investigation. Of note, only 19.6% of RLS heritability was explained by all the SNPs in this meta-analysis, suggesting that there are more risk loci to be discovered. This study shows how different data sets, with very different methods for determining disease-status, can be effectively combined if the numbers are large enough. Data from genomics companies such as 23andMe can be useful in genetic risk studies even if phenotypic information is limited and self-reported. The novel risk loci identified in this study will underpin efforts to understand better the pathophysiology of RLS.

Schormair, B. et al. (2017) Lancet Neurol 16: 898–907.

## Association of restless legs syndrome with risk of suicide and self-harm

Given the association between RLS and multiple chronic physical and mental conditions, it is unsurprising that RLS has been linked with an increase of 30–90% in all-cause mortality. However, teasing apart whether RLS itself predisposes patients to increased mortality rates, and how that risk is mediated, is difficult due to the many confounders. This paper tried to assess the increased risk of self-harm or suicide that could be directly attributed to RLS. It used a large database pertaining to commercially insured individuals in the USA to retrospectively identify a study population with RLS: those aged 20–65 with at least one claim for RLS between 2006 and 2008. Individuals with previous history of self-harm, suicide, cardiovascular disease, or cancer were excluded. The 24,179 people with a history of RLS within this timeframe were then individually matched with six controls, and the incidence of self-harm or suicide in this case–control population was then calculated for the subsequent years 2009–2014. As expected, individuals with RLS had significantly more medical comorbidities such as diabetes, obstructive sleep apnoea, and depression, and took more regular medications. They also tended to live more rurally. There were 119 recorded cases of suicide or self-harm in the total study population of 169,373 individuals followed over 5 years. There was a significantly increased risk of suicide or self-harm in the RLS group after accounting for age, sex, and multiple potential confounders such as comorbidities, medications, alcohol use, and region of residence [hazard ratio 2.66 (1.70–4.15; 95% confidence interval)]. Further sensitivity analyses showed that the increased risk of suicide or self-harm in the RLS group was maintained even if individuals with depression, obstructive sleep apnoea, or common chronic diseases were removed from the analysis.

*Comment*. This study demonstrated a robust association between RLS and self-harm or suicide in this particular data set. It analysed a very large population and used many covariates to try to control for confounders. It is unclear why RLS might be linked to self-harm or suicide, particularly as the study controlled for potential mediating factors such as depression and insomnia. The results should also be interpreted with caution as the data were based on a very small number of cases of self-harm or suicide in a retrospective, convenience data set comprising only insured individuals aged 20–65. In addition, despite efforts to control for covariates, there were still many potential confounders: for example, none of smoking status, alcohol consumption, or severity of depression was recorded in the data.

Zhuang, S. et al. (2019) JAMA Network Open 2(8):e1999966

## Risk of resistant hypertension associated with restless legs syndrome and periodic limb movements during sleep: a study on 673 treated hypertensive individuals

Hypertension has been associated with a range of sleep disorders such as obstructive sleep apnoea and insomnia with short-sleep duration. RLS is associated with periodic limb movements during sleep (PLMS) in over 80% of cases and this contributes to sleep fragmentation and deprivation, which can drive hypertension. This retrospective study attempted to show a direct association between RLS and resistant hypertension, defined as hypertension that is uncontrolled despite three antihypertensives from different classes or controlled with four agents. The starting population was 3511 adults who had undergone sleep assessments in clinic between 2002 and 2014. Of these, 673 had treated hypertension, and 272 of these met the criteria for resistant hypertension. Overall, 35.1% of this population had documented RLS. Individuals with resistant hypertension had significantly decreased sleep latency and lower mean oxygen saturations on polysomnography. Frequent RLS and PLMS (> 26 per hour) was also significantly associated with resistant hypertension [odds ratio 1.99 (1.25–3.18; 95% confidence interval)], and this association survived correction for confounders such as age, sex, comorbidities, insomnia, and BMI. Interestingly, those with less frequent RLS and PLMS did not have an increased risk of resistant hypertension in the multivariate analysis.

*Comment*. RLS is much more prevalent in hypertensive individuals than in the general population. This study showed a significant, potentially dose-dependent, association of RLS and PLMS with resistant hypertension, suggesting that the impact of RLS and PLMS on sleep might drive hypertension. This hypothesis would fit with other sleep disorders, such as obstructive sleep apnoea, being risk factors for hypertension, although the pathophysiology remains poorly understood. However, this was a small, retrospective study of hypertensive individuals who agreed to a sleep study. There were many potential confounders, and cause-and-effect could not be demonstrated.

Hein, M. et al. (2019) Sleep Medicine 63: 46–56

## Conclusion

The novel associations of RLS with self-harm and suicide and resistant hypertension discussed here reinforce the importance of prompt and effective treatment of RLS. Knowledge of genetic risk factors should improve our understanding of the underlying pathology and might enable Mendelian randomisation approaches to help disentangle the web of chronic disease associations with RLS.

